# Two Cases of Convnlsions during Dentition Arrested by Sacrification of the Gums

**Published:** 1870-04

**Authors:** G. Stevenson Smith


### ARTICLE VII.

### *Two Cases of Convulsions During Dentition Arrested by Scarification of the Gums.*

By G. Stevenson Smith, L. R. C. S. E.

Since Dr. Cairns communicated his able paper on the Scarification of the Gums to this Society, I have chanced to meet with two cases of convulsions in young children, in whom the violent and alarming excitement of the nervous system was completely allayed by lancing the gums.

A. M., aged six months, a sickly-looking infant, had not been well for a day or two, and when I was asked to see him he had much heat of skin and of the head, and had vomited several times. The pulse was sharp and quick, and for twenty-four hours there had been numerous successive attacks of general convulsions. Failing to find any cause for the fits in the state of the general health, I examined the mouth, and found the lower gum red, tumid, and glistening. I divided its tense margin with a lancet, and the little patient appeared to get immediate relief. At my visit next day, I found him lively and contented, the temperature had fallen, the gastric irritation had subsided, and there had been no more convulsions.

L. S.; aged eight months, had been fretting much for some days, was hot and restless at night, had a burning head, quick pulse, and a ceaseless whining cry. I found that he had repeated attacks of convulsions, and when I arrived he was in a state of opisthotonos, this condition having existed for several hours. Having carefully examined the child, I found nothing to account for the nervous symptoms, save that the upper gum was hot, red, and swollen. He had cut the two lower incisors. I drew the point of a lancet across the tumid gum, and next day I found that the opisthotonos had passed away very soon after the operation. There were no more fits, and the child was comparatively well. The two upper incisors made their appearance in two days, and when I saw the child the other day hc was in perfect health.

Similar cases I have frequently met with before, and the members of the Society must have had the same experience

In Dr. Cairn's paper three questions were put, which I shall now endeavour to answer *seriatim.*

1*st*, Does sacrification do any good? Does it relieve local pain or prevent and arrest convulsions, laryngismus stridulus, diarrhœa, etc.? To this I reply in the affirmative. It does relieve local pain in many a case, and how this can be doubted for a moment I am at a loss to understand. The little patient cannot speak, says Dr. Cairns, and how can you be sure that you have given relief? It seems to me that, if we cannot interpret the feelings of a little child because it has not yet acquired the use of articulate speech, we are not well fitted to treat the diseases incident to infancy, and have yet to cultivate a most important part of our professional education. The simple wagging of a dog's tail conveys to his master a clear and distinct expression of the feelings whitch animate his canine breast ;and do not the calm repose, the sparkling eye, the joyful crowing of our little patients manifest their relief from suffering as decidedly as the sleeplessness, the fretfulness, and the shrill cry of pain tell of discomfort and distress? But Dr. Cairns does not believe that by abstracting blood from an inflamed part you can in the least degree either reduce or modify the inflammation. The part, he says, continues to be as red, as hot, and as painful as before. Such ideas are only to be explained on the supposition that our friend never practices local depletion, and is consequently a stranger to the beneficial effects of such a remedy. Has he never seen relief following the opening of an abscess, or the application of leeches to a swelled testicle or to the belly in a case of acute peritonitis? If he has not, then I can easily comprehend why he doubts that the abstraction of a little blood from a congested gum can alleviate pain.

That scarification may prevent and arrest convulsions I firmly believe, and in this opinion I know that I am supported by a perfect cloud of witnesses. Dr. Brown-Sequard has shown how easy it is by pinching or otherwise irritating certain nervous filaments in the guinea-pig to induce convulsions; and I think one can without difficulty understand how irritation of the branches of the fifth pair may produce convulsions in infants whose nervous system is so susceptible of impressions That the convulsions in my two cases were caused in this way, and that they were arrested by relieving the congested gums, I have not the faintest shadow of a doubt. Dr. Cairns may say that the cessation of the attacks following upon scarification was a mere matter of coincidence and nothing more, and that the convulsions might have disappeared even suppose nothing had been done. This I do not deny; but I am inclined to think that, instead of ceasing spontaneously, there was a much greater probability that they would have continued. Besides, this is not, in my opinion, the proper spirit in which one should discuss the influence of any remedial measure. The progress of medical and all other science is no doubt furthered by a certain amount of wholesome scepticism, but surely it must be retarded if we doubt everything and believe nothing. As was well remarked by Dr. James Young, in a previous discussion on this subject—"I do not consider myself a heroic practitioner in any sense of the term, but at the same time I have no sympathy with those who stand idly by when something ought to be done. There is a great deal of truth and a spice of grim humour in the remark of one of the fathers of medicine, that the expectant treatment of disease is "a meditation upon death." And I think it is highly culpable to refuse to perform so trifling an operation as scarification of the gums when we are convinced that it is in them that the source of the irritation resides.

Dr. Cairn's second question was, Does scarification do any harm? To this I reply that, so far as my experience goes, it does not. Indiscriminate lancing of the gums cannot but be productive of mischief, but in properly selected cases I believe the operation is never followed by any evil consequences. That it may occasionly lead to fatal hæmorrhage I cannot deny; but such cases are extremely rare, and can only be regarded as accidents, against which it is almost impossible to provide. The extraction of a tooth may lead to death in the same way, but no one should on that account denounce the operation as an unjustifiable one. Besides, as Dr. Ritchie suggested, the existance of the hæmorrhagic diathesis might be ascertained by inquiry as to the history of the vaccination.

Dr. Cairns third question was, Is scarification in the circumstances warrantable? He thinks it is not, because it inflicts unnecesary pain, superinduces some of those conditions which it professes to remedy, and, at the best, is a mere experiment. In regard to the first mentioned reasons, I have nothing further to say than merely to repeat what I have stated already, that in properly selected cases no such objection can be for a moment entertained. But he says scarification is at the best an experiment. Now, by an experiment I understand something that is done in order to discover an uncertain or unknown effect. But the effect of scarification is neither unknown nor uncertain, and therefore scarification cannot properly be called an experiment.

We know positively that irritation of a nerve trunk may induce convulsions, and in dentition how very often do we find the trifacial excited by inflammation of the gum. The lancing relieves congestion, tension, and pain, and by allaying irritation prevents or arrests convulsions. Such, at all events, is my belief—a belief which the experience of my seniors tends to strengthen and confirm.—*Edinburg Journal Canada Medical Journal.*

